# Deletion of the eIF2α Kinase GCN2 Fails to Rescue the Memory Decline Associated with Alzheimer’s Disease

**DOI:** 10.1371/journal.pone.0077335

**Published:** 2013-10-11

**Authors:** Latha Devi, Masuo Ohno

**Affiliations:** 1 Center for Dementia Research, Nathan Kline Institute, Orangeburg, New York, United States of America; 2 Department of Psychiatry, New York University Langone Medical Center, New York, New York, United States of America; Federal University of Rio de Janeiro, Brazil

## Abstract

Emerging evidence suggests that dysregulated translation through phosphorylation of eukaryotic initiation factor-2α (eIF2α) may contribute to Alzheimer’s disease (AD) and related memory impairments. However, the underlying mechanisms remain unclear. Here, we crossed knockout mice for an eIF2α kinase (GCN2: general control nonderepressible-2 kinase) with 5XFAD transgenic mice, and investigated whether GCN2 deletion affects AD-like traits in this model. As observed in AD brains, 5XFAD mice recapitulated significant elevations in the β-secretase enzyme BACE1 and the CREB repressor ATF4 concomitant with a dramatic increase of eIF2α phosphorylation. Contrary to expectation, we found that GCN2^−/−^ and GCN2^+/−^ deficiencies aggravate rather than suppress hippocampal BACE1 and ATF4 elevations in 5XFAD mice, failing to rescue memory deficits as tested by the contextual fear conditioning. The facilitation of these deleterious events resulted in exacerbated β-amyloid accumulation, plaque pathology and CREB dysfunction in 5XFAD mice with GCN2 mutations. Notably, GCN2 deletion caused overactivation of the PKR-endoplasmic reticulum-related kinase (PERK)-dependent eIF2α phosphorylation pathway in 5XFAD mice in the absence of changes in the PKR pathway. Moreover, PERK activation in response to GCN2 deficiency was specific to 5XFAD mice, since phosphorylated PERK levels were equivalent between GCN2^−/−^ and wild-type control mice. Our findings suggest that GCN2 may be an important eIF2α kinase under the physiological condition, whereas blocking the GCN2 pathway under exposure to significant β-amyloidosis rather aggravates eIF2α phosphorylation leading to BACE1 and ATF4 elevations in AD.

## Introduction

Alzheimer’s disease (AD) is a devastating neurodegenerative disorder and represents the most common form of dementia among the elderly. Although the molecular cause of AD has not been completely understood, recent evidence increasingly implicates the aberrant translational machinery through eukaryotic initiation factor-2α (eIF2α) in the pathogenesis of this disease. It has been reported that eIF2α phosphorylation is significantly increased in brains of AD patients and different lines of amyloid precursor protein (APP) transgenic mice [1]–[6]. While phosphorylation of eIF2α at Ser51 inhibits general translation initiation, it paradoxically causes translational activation of a subset of mRNAs that contain upstream open reading frames. Those molecules include the β-secretase called β-site APP-cleaving enzyme 1 (BACE1) [2], [3] and the transcriptional modulator activating transcription factor 4 (ATF4) [7], [8]. Consistent with increases in phosphorylated eIF2α, protein and activity levels of BACE1, a key enzyme responsible for initiating the production of β-amyloid (Aβ) peptides, are highly elevated in AD brains [9]–[11]. Moreover, a recent report also demonstrates AD-related upregulation of ATF4 [12], which is known as a repressor of cAMP response element binding protein (CREB)-dependent transcription critical for memory consolidation (CREB-2) [13], [14]. Therefore, these findings suggest that overactivation of the eIF2α phosphorylation pathway may account for Aβ accumulation and cognitive impairments in AD by accelerating β-amyloidogenesis through BACE1 elevations and directly suppressing CREB function. However, little is known about signaling mechanisms that may trigger the eIF2α-mediated translational dysregulation associated with AD.

The phosphorylation of eIF2α is controlled by four protein kinases such as general control nonderepressible-2 kinase (GCN2), double-stranded RNA-activated protein kinase (PKR), PKR-endoplasmic reticulum-related kinase (PERK) and heme-regulated inhibitor kinase (HRI) [15], [16]. The eIF2α kinases except for HRI are prominently expressed in the mammalian brain [15]–[17]. In particular, GCN2 is enriched in the hippocampus, mediates eIF2α phosphorylation, and regulates synaptic and mnemonic functions under the physiological condition [18]. However, it remains to be examined whether the GCN2 pathway may contribute to the pathophysiology of AD and related memory impairments. To address this question, we crossed GCN2-deficient mice with the 5XFAD transgenic mouse model of AD. Here, we report that GCN2 deletion unexpectedly facilitates elevations in BACE1 and ATF4 expression, failing to rescue memory deficits in 5XFAD mice. Interestingly, we found that the exacerbation of these pathogenic events is accompanied by overactivation of the PERK-eIF2α phosphorylation pathway and leads to accelerated β-amyloidogenesis and CREB dysfunction in GCN2-deficient 5XFAD mice. Our results suggest that PERK plays a pivotal role in mediating eIF2α phosphorylation responsible for BACE1 and ATF4 elevations associated with β-amyloidosis and that the GCN2 pathway may function as a negative regulator of PERK-dependent eIF2α phosphorylation under AD conditions.

## Materials and Methods

### Animals

We used 5XFAD mice (Tg6799 line) that co-overexpress familial AD (FAD) mutant forms of human APP (the Swedish mutation: K670N, M671L; the Florida mutation: I716V; the London mutation: V717I) and presenilin 1 (PS1) (M146L and L286V mutations) transgenes under transcriptional control of the neuron-specific Thy-1 promoter [19]–[21]. Hemizygous 5XFAD transgenic mice (C57BL/6 background) were crossbred to homozygous GCN2 knockout mice (C57BL/6 background) [7], [18]. The resultant F1 progeny were intercrossed, yielding animals with the genotypes of interest. Genotyping was performed by PCR analysis of tail DNA. All experiments were done blind with respect to the genotype, using the mice in the F2 progeny. According to accelerated Aβ42 production due to a combination of five FAD mutations, 5XFAD mice begin to develop visible amyloid deposition as early as 2 months of age and exhibit memory declines on hippocampus-dependent behavioral tasks between 4 and 6 months concomitant with moderate Aβ accumulation and impaired synaptic physiology at Schaffer collateral-CA1 pathways [19], [20], [22]–[24]. In this study, we investigated the effects of GCN2 deletion in 5XFAD mice at 8–9 months of age, which show significant BACE1 elevations associated with robust plaque deposition [25]–[28] as observed in human AD brains [9]–[11]. Since our previous study shows that there is no sex difference in cerebral Aβ levels in 5XFAD mice except for the younger age (≤3 months) (Oakley et al., 2006), a similar number of males and females were used for the experiments. All animal procedures were conducted in accordance with National Institutes of Health guidelines and were approved by the Nathan Kline Institute Animal Care and Use Committee (Assignment Number: AP2011-390).

### Contextual Fear Conditioning

Contextual fear conditioning was tested as described previously [22], [29], [30]. In this behavioral assay, mice learn to associate a distinct context (CS: conditioned stimulus) with aversive footshocks (US: unconditioned stimulus) through hippocampus-dependent mechanisms [31], [32]. The experiments were performed using four standard conditioning chambers, each of which was housed in a soundproof isolation cubicle and equipped with a stainless-steel grid floor connected to a solid-state shock scrambler. Each scrambler was connected to an electronic constant-current shock source that was controlled via an interface connected to a Windows XP computer running FreezeFrame software (Coulbourn Instruments, Allentown, PA, USA). A digital camera was mounted on the steel ceiling of each chamber, and video signals were sent to the same computer for analysis. During training, mice were placed in the conditioning chamber for 3 min and then received two footshocks (1.0 mA, 2 s) at a 1-min interval. After the last shock delivery, mice were left in the chamber for another 30 s. Contextual fear memory was evaluated by scoring freezing behavior (the absence of all movement except for that needed for breathing) for 3 min when the mice were placed back into the same conditioning chamber 24 h after training. The automated FreezeFrame system (Coulbourn Instruments) was used to score the amount of freezing. After behavioral testing, some mice were sacrificed for immunoblotting/ELISA experiments and others were perfused for immunohistochemistry.

### Immunoblot Analysis

Hippocampal samples were taken from the mice under deep isoflurane anesthesia and were snap-frozen for biochemical assays. For western blot analysis, each sample was homogenized in 5 volumes of modified RIPA buffer containing 150 mM NaCl, 50 mM Tris HCl (pH 8.0), 1 mM EDTA, 1% IGEPAL, 0.5% sodium deoxycholate, 0.1% SDS and protease/phosphatase inhibitor cocktail (Calbiochem, La Jolla, CA, USA), and centrifuged at 10,000 g for 10 min to remove any insoluble material. Protein concentrations were determined by a BCA protein assay kit (Pierce, Rockford, IL, USA), and 10–50 µg of protein was run on NuPAGE 4–12% Bis-Tris gels or 7% Tris-Acetate gels (Invitrogen, Carlsbad, CA, USA) and transferred to nitrocellulose membrane. After blocking, membranes were probed with the following primary antibodies: anti-BACE1 (1∶1,000, B0681, Sigma-Aldrich, St. Louis, MO, USA), an antibody that recognizes C-terminal epitope in APP (1∶1,000, C1/6.1, kindly provided by Dr. Paul Mathews, Nathan Kline Institute) to detect full-length APP/C-terminal fragments, anti-phospho-eIF2α (Ser51) (1∶1,000, #3398, Cell Signaling Technology, Danvers, MA, USA), anti-eIF2α (1∶2,000, #9722, Cell Signaling Technology), anti-phospho-PERK (Ser713) (1∶1,000, #649401, BioLegend, San Diego, CA), anti-PERK (1∶2,000, #3192, Cell Signaling Technology), anti-phospho-PKR (Thr451) (1∶500, 07-886, Millipore, Billerica, MA, USA), anti-ATF4 (1∶2,000, 10835-1-AP, Proteintech, Chicago, IL, USA), anti-phospho-CREB (Ser133) (1∶1,000, #9198, Cell Signaling Technology), anti-CREB 1∶2,000, #9197, Cell Signaling Technology) and anti-β-actin (1∶15,000, AC-15, Sigma-Aldrich). They were then incubated with horseradish peroxidase-conjugated secondary IgG. Immunoblot signals were visualized by an ECL chemiluminescence substrate reagent kit (Pierce), and were quantified by densitometric scanning and image analysis using Quantity One software (Bio-Rad Laboratories, Hercules, CA, USA).

### ELISAs of Aβ40 and Aβ42

Sandwich Aβ ELISAs were performed as described previously [3], [30], [33]. Briefly, each hippocampal sample was extracted in 8X cold 5 M guanidine HCl plus 50 mM Tris HCl (pH 8.0) buffer, and centrifuged at 20,000 g for 1 h at 4°C to remove insoluble material. Final guanidine HCl concentrations were below 0.1 M. Protein concentrations were determined by a BCA kit (Pierce). To quantitate total levels of cerebral Aβ40 and Aβ42, supernatant fractions were analyzed by a well-established human Aβ40 and Aβ42 ELISA kits (KHB3481 and KHB3441, Invitrogen), respectively, according to the protocol of the manufacturer. Optical densities at 450 nm of each well were read on a VersaMax tunable microplate reader (Molecular Devices, Sunnyvale, CA, USA), and sample Aβ40 and Aβ42 concentrations were determined by comparison with the respective standard curves. Aβ40 and Aβ42 concentration values were normalized to total protein concentrations and expressed as the percentage of 5XFAD mouse controls.

### Double Immunofluorescence Labeling

Mice were transcardially perfused with 0.1 M phosphate buffered saline (PBS, pH 7.4) followed by 4% paraformaldehyde in PBS under deep isoflurane anesthesia. Brains were post-fixed for 24 h in 4% paraformaldehyde in PBS at 4°C and transferred to PBS. The brain was sectioned coronally at 30 µm using a vibratome (VT1200, Leica Microsystems, Wetzlar, Germany). Successive sections were taken at levels between –1.7 and –1.9 mm to bregma according to the mouse brain atlas of Franklin and Paxinos [34] and stored in PBS containing 0.05% sodium azide at 4°C. After the sections were permeabilized with 0.3% Triton X-100, they were incubated overnight at 4°C with the rabbit monoclonal antibody against BACE1 (1∶200, #5606, Cell Signaling Technology) and Alexa Fluor 488 labeled monoclonal anti-Aβ1–16 (6E10) antibody (1∶200, SIG-39347; Covance, Princeton, NJ, USA). Immunofluorescence labeling of anti-BACE1 was performed by a 1-h reaction with Alexa Fluor 594 conjugated anti-rabbit IgGs (1∶750, Invitrogen) at room temperature. The sections were then washed three times in PBS and mounted with anti-fading medium. Control sections were processed with the omission of the primary antibody in the incubation buffer, and these controls yielded no specific labeling in brain sections. Immunostained sections were imaged with a confocal fluorescence microscope (LSM 510 Meta, Zeiss, Oberkochen, Germany) with a 40X objective.

### Aβ Immunohistochemistry

The brain sections were stained by the avidin-biotin peroxidase complex method as described previously [27], [30], [35]. Briefly, the sections were incubated overnight at 4°C with mouse monoclonal anti-Aβ1–16 (6E10) antibody (1∶200, SIG-39347; Covance). The ABC kit (PK-2200; Vector Laboratories, Burlingame, CA, USA) was utilized with 3,3′-diaminobenzidine tetrahydrochloride as a chromogen to visualize the reaction product. The sections were then mounted on charged slides, dehydrated in a series of alcohol, cleared in xylene and covered with a coverslip. Light microscopy was conducted on an Axioskop 2 microscope equipped with an AxioCaM HRc digital camera (Zeiss) for capturing images. Semi-quantitative analysis was performed using AxioVision imaging software with the AutoMeasure module (Zeiss). Identified objects after thresholding were individually inspected to confirm the object as a plaque or not in a blinded manner. Percentage area occupied by Aβ deposits in the hippocampus was assessed bilaterally to compare plaque burden between 5XFAD control and GCN2^−/−^·5XFAD mice.

### Data Analysis

Significant differences between the groups were determined by a one-way or two-way ANOVA and *post-hoc* Fisher’s PLSD tests were applied following all ANOVAs showing significance. Data were presented as mean ± SEM and the level of significance was set for *p* value less than 0.05.

## Results

### GCN2 Deficiency Aggravates BACE1 Elevation and β-amyloidogenesis in 5XFAD Mice

**Figure 1:**
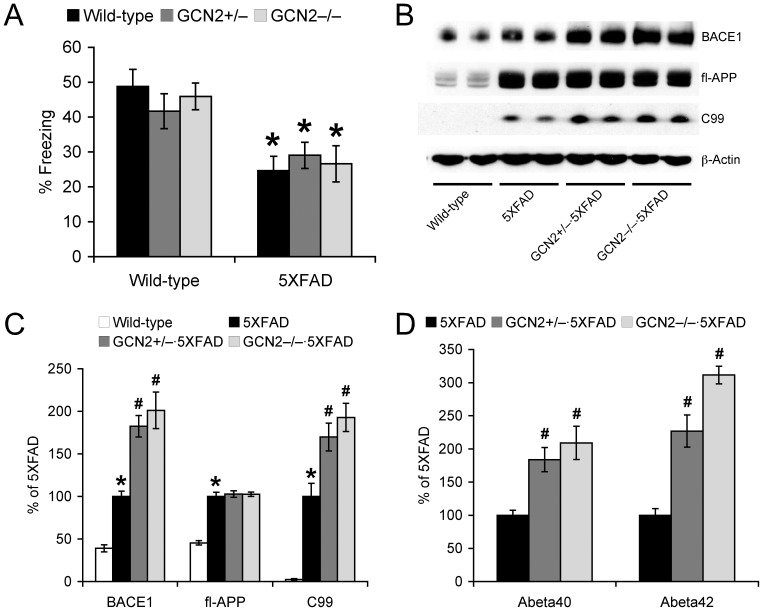
Effects of GCN2 deletion on memory deficits and β-amyloidogenic processing of APP in 5XFAD mice. (A) GCN2^−/−^ and GCN2^+/−^ deficiencies had no effects on memory impairments in 5XFAD mice (*p<0.05 vs. wild-type), as tested by the contextual fear conditioning (*n* = 11–19). (B) Representative immunoblots of protein extracts from hippocampal homogenates of mice. (C) Immunoreactive bands were quantified and expressed as the percentage of 5XFAD control mice (*n* = 5–8). Note that GCN2 mutations further increase BACE1 and C99 levels without affecting APP overexpression in 5XFAD mice (*p<0.05 vs. wild-type, ^#^ *p*<0.05 vs. 5XFAD). (D) Levels of total Aβ40 and Aβ42 were quantified by sandwich ELISAs of guanidine extracts of hippocampal samples and expressed as the percentage of 5XFAD controls (*n* = 5–10). GCN2 mutations also significantly elevate Aβ40 and Aβ42 concentrations in 5XFAD mice (^#^ *p*<0.05 vs. 5XFAD). All data are presented as mean ± SEM.

We first tested whether GCN2^−/−^ and GCN2^+/−^ gene deletion may rescue memory deficits in 5XFAD mice, using the hippocampus-dependent contextual fear conditioning paradigm (Fig. 1A). Wild-type control mice exhibited a robust conditioned fear response as assessed by freezing (the absence of all but respiratory movements) when placed back into the conditioning chamber 24 h after training with two CS-US pairings. A two-way ANOVA for contextual freezing revealed a significant main effect of 5XFAD genotype (*p*<0.05) in the absence of a main effect of GCN2 mutations or a significant 5XFAD X GCN2 interaction. *Post-hoc* Fisher’s PLSD tests showed that freezing levels were significantly reduced in 5XFAD mice irrespective of the presence of GCN2 mutations, as compared with wild-type controls (*p*<0.05). Therefore, neither GCN2^−/−^ nor GCN2^+/−^ deletion affected memory impairments in 5XFAD mice. Furthermore, freezing levels were indistinguishable between GCN2^−/−^, GCN2^+/−^ and wild-type mice, demonstrating that GCN2 deletion did not affect baseline memory performances on the wild-type background.

To clarify the mechanisms underlying behavioral failure to ameliorate memory deficits in 5XFAD mice, we next performed immunoblot analysis of hippocampal samples and examined the effects of GCN2 deficiency on the β-amyloidogenic processing of APP (Fig. 1B). Contrary to expectation, a one-way ANOVA followed by *post-hoc* Fisher’s PLSD tests indicated that GCN2^−/−^ and GCN2^+/−^ deficiencies exacerbated elevations of BACE1 expression in 5XFAD mice (*p*<0.05) (Fig. 1C). In accordance with these changes, GCN2^−/−^ and GCN2^+/−^ mutations further elevated levels of the β-cleaved C-terminal fragment of APP (C99) (*p*<0.05) without affecting APP overexpression in 5XFAD mice. Moreover, sandwich ELISAs showed that hippocampal Aβ40 and Aβ42 levels were significantly increased in GCN2^−/−^·5XFAD and GCN2^+/−^·5XFAD mice as compared with those of 5XFAD control mice (*p*<0.05) (Fig. 1D).

**Figure 2:**
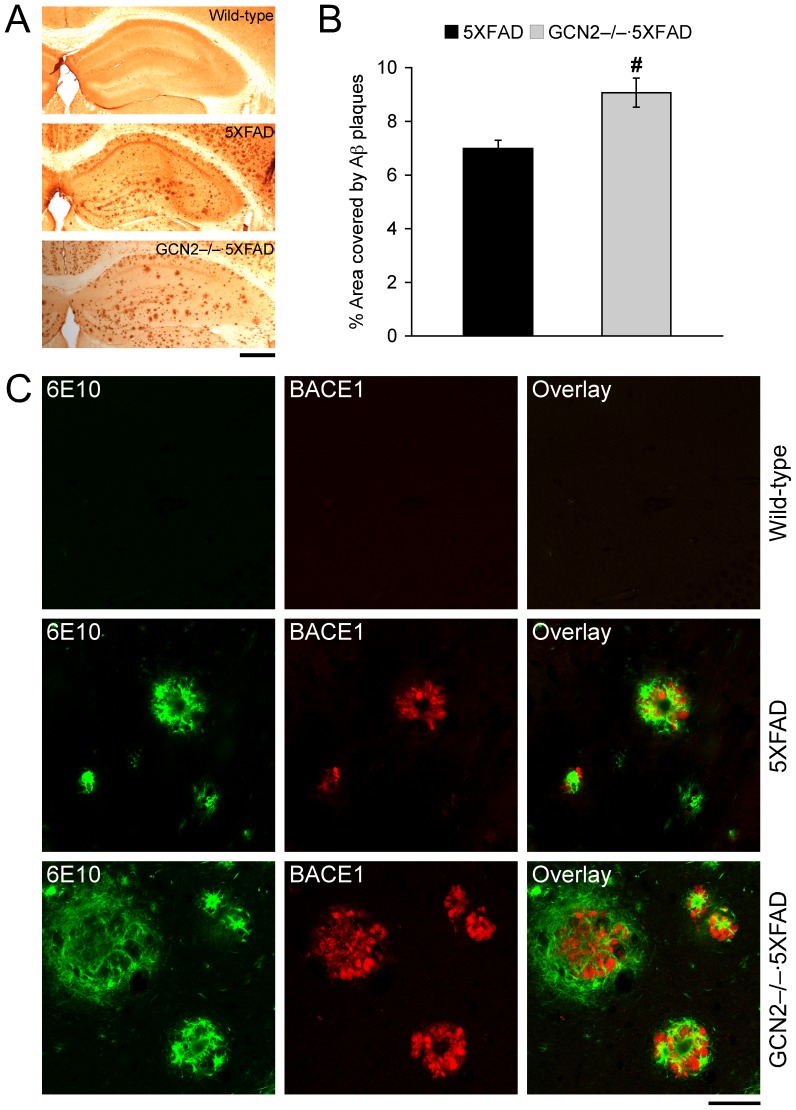
Effects of GCN2 deletion on Aβ load and plaque-associated elevation of BACE1 expression in 5XFAD mice. (A) Brain sections were immunostained with the 6E10 anti-Aβ antibody. Shown are representative photomicrographs of the hippocampal region. Scale bar = 500 µm. (B) Percentage area occupied by Aβ deposits in the hippocampus was measured for quantification (*n* = 6–8). GCN2^−/−^ deletion exacerbates plaque burden in 5XFAD mice (^#^ *p*<0.05 vs. 5XFAD). Data are presented as mean ± SEM. (C) Double immunofluorescence labeling with 6E10 anti-Aβ (green) and anti-BACE1 (red) antibodies (*n* = 3–4). Note the high degree of colocalization of Aβ and BACE1 immunoreactivities in the hippocampus of 5XFAD and GCN2^−/−^·5XFAD mice. Scale bar = 50 µm.

In agreement with ELISA data, Aβ immunostaining with 6E10 antibody revealed that hippocampal plaque load in GCN2^−/−^·5XFAD mice was significantly greater than that of 5XFAD control mice (*p*<0.05) (Fig. 2A, 2B). We further performed double immunofluorescence labeling to investigate the association between Aβ deposition and BACE1 expression in the hippocampus (Fig. 2C). As reported previously [3], [25], [26], [28], BACE1 immunoreactivity was colocalized with amyloid plaques in 5XFAD mice. Furthermore, we observed that more extensive immunoreactivity for BACE1 was highly associated with increased levels of Aβ deposits in GCN2^−/−^·5XFAD mice. Therefore, these results suggest that GCN2^−/−^ deficiency may aggravate a positive feedback pathogenic link between Aβ plaque formation and BACE1 elevation in 5XFAD mice.

### GCN2 Deficiency Overactivates PERK-dependent eIF2α Phosphorylation in 5XFAD Mice

**Figure 3:**
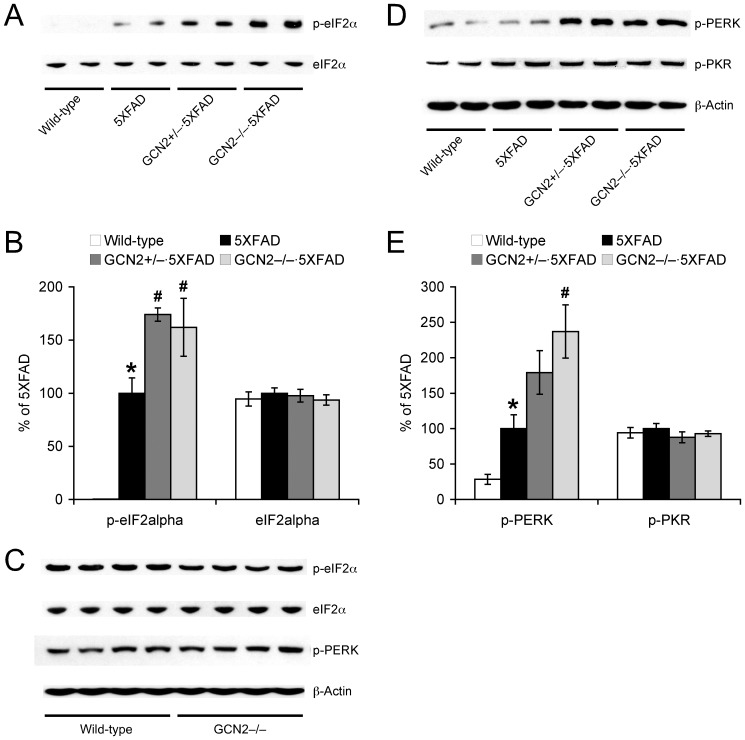
Effects of GCN2 deletion on eIF2α phosphorylation pathways in 5XFAD mice. (A, C, D) Representative immunoblots of protein extracts from hippocampal homogenates of mice. (B, E) Immunoreactive bands were quantified and expressed as the percentage of 5XFAD control mice (*n* = 5–12). Note that GCN2 deficiencies aggravate eIF2α phosphorylation without affecting total eIF2α levels in 5XFAD mice. Moreover, GCN2 deletion induces overactivation of another eIF2α kinase PERK in 5XFAD mice, while PKR activities are not changed. *p<0.05 vs. wild-type, ^#^ *p*<0.05 vs. 5XFAD. All data are presented as mean ± SEM.

To address the mechanisms by which GCN2 deletion exacerbated rather than suppressed BACE1 elevations and β-amyloidogenesis in 5XFAD mice, we investigated eIF2α phosphorylation pathways (Fig. 3). Consistent with recent data from our laboratory and others [2]–[4], BACE1 elevation was closely associated with a robust increase of eIF2α phosphorylation in the hippocampus of 5XFAD mice (*p*<0.05) (Fig. 3A, 3B). Interestingly, we found that GCN2^−/−^ and GCN2^+/−^ mutations further elevated levels of phosphorylated eIF2α (*p*<0.05) without affecting total eIF2α levels in 5XFAD mice, suggesting the acceleration of eIF2α phosphorylation-dependent translational upregulation of BACE1 in these mice. In contrast, eIF2α phosphorylation was reduced in GCN2^−/−^ mice as compared with that of wild-type controls (Fig. 3C), supporting a previous finding that GCN2 is a major kinase responsible for mediating eIF2α phosphorylation on the wild-type background [18].

We further explored whether other eIF2α kinases may be affected in GCN2^−/−^·5XFAD and GCN2^+/−^·5XFAD mice (Fig. 3D, 3E). In accordance with changes in phosphorylated eIF2α levels, activation of the PERK pathway, as measured by an increase in phosphorylated PERK, was significantly facilitated by GCN2^−/−^ deletion in 5XFAD mice (*p*<0.05); a trend toward facilitation was observed in GCN2^+/−^·5XFAD mice (*p = *0.1). In contrast, there was no change in levels of phosphorylated PKR, another eIF2α kinase, between the four groups of mice. Importantly, levels of phosphorylated PERK were indistinguishable between GCN2^−/−^ and wild-type control mice (Fig. 3C), indicating that PERK activation in response to GCN2 deficiency is specific to 5XFAD mice. Collectively, these data suggest that overactivation of the PERK pathway may account for the mechanisms by which GCN2 deletion under ER stress may aggravate eIF2α phosphorylation-dependent translational upregulation of BACE1 expression and consequently facilitate β-amyloidogenesis in 5XFAD mice.

### GCN2 Deficiency Aggravates ATF4 Elevation and CREB Dysfunction in 5XFAD Mice

**Figure 4:**
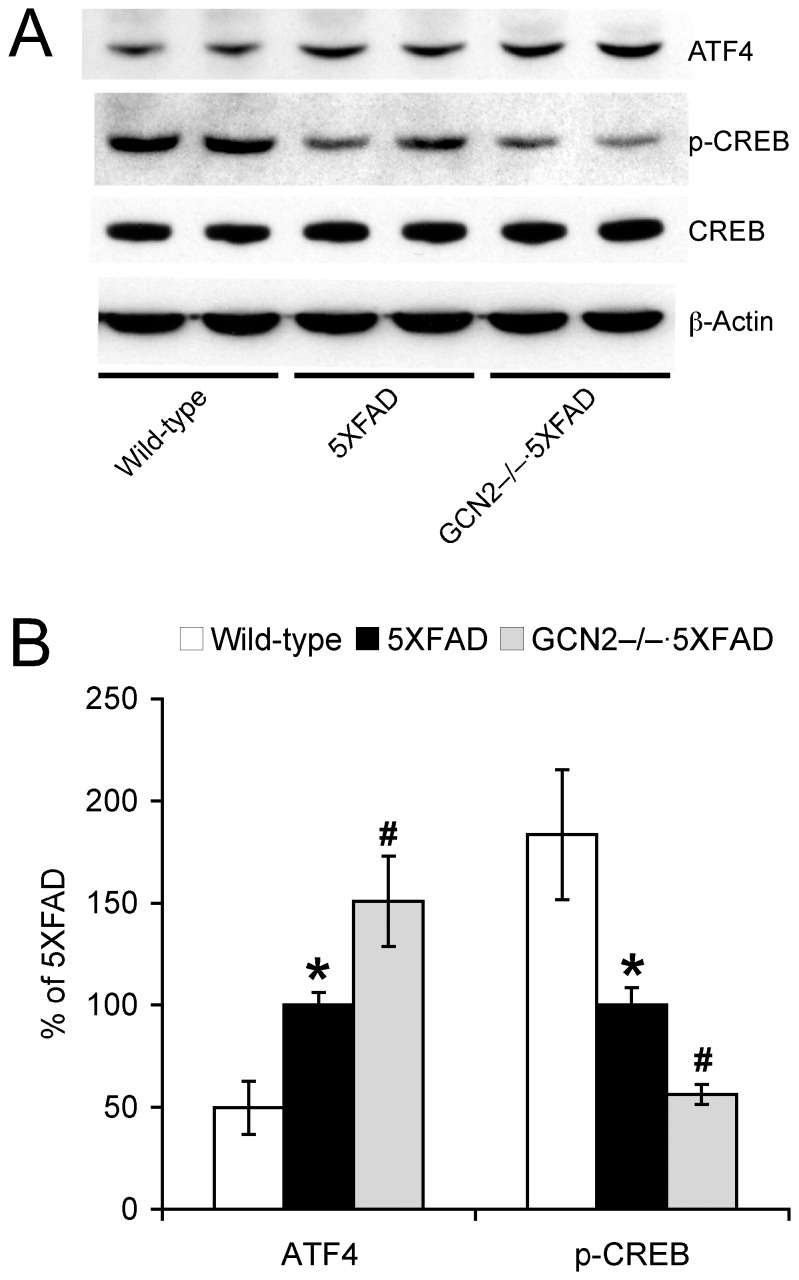
Effects of GCN2 deletion on ATF4 and CREB pathways in 5XFAD mice. (A) Representative immunoblots of protein extracts from hippocampal homogenates of mice. (B) Immunoreactive bands were quantified and expressed as the percentage of 5XFAD control mice (*n* = 6–8). Note that GCN2^−/−^ deletion further increases ATF4 expression and accordingly reduces levels of phosphorylated CREB without affecting total CREB in 5XFAD mice. *p<0.05 vs. wild-type, ^#^ *p*<0.05 vs. 5XFAD. All data are presented as mean ± SEM.

Since eIF2α phosphorylation is known to induce translational elevation of ATF4 in addition to BACE1, we conducted immunoblot analysis to determine whether GCN2 gene deletion may also affect ATF4 expression and CREB signaling in 5XFAD mice (Fig. 4A). Consistent with changes in phosphorylated eIF2α levels (Fig. 3A, 3B), GCN2^−/−^ mutation enhanced rather than suppressed ATF4 elevations in the hippocampus of 5XFAD mice (Fig. 4B). Moreover, GCN2^−/−^ deletion exacerbated reductions of phosphorylated CREB without affecting total CREB levels in 5XFAD mice. Together, the results suggest that GCN2 deficiency aggravates ATF4-dependent CREB dysfunction as well as BACE1 elevation associated with β-amyloidosis in 5XFAD mice, most likely by causing further activation of another eIF2α kinase PERK.

## Discussion

It has been demonstrated that the eIF2α kinase GCN2 is enriched in the hippocampus, where GCN2^−/−^ deletion is able to reduce levels of phosphorylated eIF2α on the wild-type background [18]. Therefore, GCN2 is a crucial eIF2α kinase that is responsible for eIF2α phosphorylation under the physiological condition. In this study, we tested the hypothesis that genetic deletion of GCN2 may also reduce eIF2α phosphorylation that overly occur in brains of AD patients and a battery of APP transgenic mouse models [1]–[6], and thereby exert some beneficial effects on AD-like traits such as β-amyloidosis, CREB dysfunction and memory deficits. Contrary to expectation, we found that not only GCN2^−/−^ but also GCN2^+/−^ mutation facilitates rather than suppresses eIF2α phosphorylation in the hippocampus of 5XFAD mice that suffer from massive Aβ plaque pathology. Our results clearly indicate that signaling mechanisms controlling eIF2α phosphorylation are different between normal and AD conditions, under which the GCN2 pathway is not a direct mediator of this detrimental event associated with translational dysregulation.

The phosphorylation of eIF2α is reported to underlie the translational upregulation of BACE1 in advanced stages of 5XFAD mice and AD patients [2]–[4]. Furthermore, we previously showed that the increase of eIF2α phosphorylation induced by Sal 003, a specific inhibitor of its phosphatase, can elevate BACE1 protein levels in 6-month-old 5XFAD mice, which have not yet showed BACE1 upregulation consistent with only marginal changes in eIF2α phosphorylation [3]. In the current study, we found that the facilitation of hippocampal eIF2α phosphorylation in GCN2^−/−^·5XFAD and GCN2^+/−^·5XFAD mice (8–9-month-old) exacerbates elevations in BACE1 expression, resulting in the acceleration of β-amyloidogenesis as evidenced by increased levels of C99 fragments, Aβ40 and Aβ42 peptides, and amyloid plaque load. Moreover, as seen in AD brains [12], 5XFAD mice showed elevated expression of ATF4, a repressor of CREB (CREB-2). We also found that GCN2 deletion aggravates CREB dysfunction, at least in part, through enhancing the eIF2α phosphorylation-dependent upregulation of ATF4 in 5XFAD mice. This is in sharp contrast with the previous observation that GCN2^−/−^ deficiency in wild-type mice downregulates the translation of ATF4 mRNA concomitant with reduced eIF2α phosphorylation, and consequently enhances CREB activity [18]. Therefore, these findings raise the possibility that the functional role of GCN2 may switch from a pivotal eIF2α kinase under the physiological condition to a negative regulator of eIF2α phosphorylation under exposure to robust β-amyloidosis in 5XFAD mice.

Recent work indicates the importance of translational control through eIF2α phosphorylation in learning and memory [15]–[17]. It has been demonstrated that genetic or pharmacologic stimulation of eIF2α phosphorylation impairs hippocampus-dependent contextual memory consolidation through upregulation of ATF4 and consequent CREB dysfunction [36], [37]. Conversely, it is reported that behavioral training for contextual fear conditioning results in reduced eIF2α phosphorylation at Ser51 [37]. Indeed, the suppression of eIF2α phosphorylation in knock-in mice with an eIF2α^+/S51A^ point mutation enhances contextual and spatial memory consolidation concomitant with ATF4 reduction [37]. Similar mnemonic facilitation is also found in GCN2^−/−^ knockout mice after weak training for the Morris water maze [18]. Therefore, these findings provide convergent evidence that eIF2α phosphorylation-dependent elevation of ATF4 negatively regulates memory consolidation processes through suppressing CREB activity under the physiological condition. In accordance with this scenario, 5XFAD mice showed impaired contextual fear conditioning that was accompanied by ATF4 elevation and CREB dysfunction in the present study. However, GCN2^−/−^ and GCN2^+/−^ deletion in 5XFAD mice unexpectedly aggravated the eIF2α phosphorylation-associated ATF4/CREB dysregulation as well as β-amyloidogenesis, thus failing to rescue the impairment of contextual learning and memory in this AD model.

To elucidate the mechanisms by which GCN2 deficiency facilitates rather than suppresses eIF2α phosphorylation in 5XFAD mice, we studied changes in other eIF2α kinase-dependent pathways. Intriguingly, we found that PERK, but not PKR, is overactivated by GCN2 deletion in 5XFAD mice in accordance with the facilitation of eIF2α phosphorylation. It is important to note that GCN2^−/−^ mutation does not induce compensatory PERK activation and is able to reduce eIF2α phosphorylation on the wild-type background. Moreover, double immunofluorescence staining showed that elevated BACE1 expression is closely associated with Aβ plaque deposition in 5XFAD mice and that this pathogenic link is aggravated in GCN2-deficient 5XFAD mice. Therefore, the results indicate that overactivation of the PERK-eIF2α pathway and further BACE1 upregulation in response to GCN2 deletion occurs specifically under exposure to significant amyloid pathology in 5XFAD mice.

PERK is primarily activated by the accumulation of misfolded protein in the endoplasmic reticulum (ER), a phenomenon called ER stress [16]. It is widely accepted that Aβ accumulation in AD brains activates PERK-dependent eIF2α phosphorylation, which inhibits the initiation of general translation and in this manner can alleviate ER stress by reducing the amount of protein transport into the ER [38], [39]. Meanwhile, the prolonged activation of ER stress-associated events in AD is thought to accelerate pathologic lesions [40], [41]. This study shows that ER stress also accompanies translational elevations of BACE1/ATF4 expression and thus aggravates Aβ neuropathology and CREB dysfunction in 5XFAD mice. It seems likely that blocking the GCN2 pathway may rather overactivate PERK-eIF2α signaling and exacerbate BACE1/ATF4 elevations under ER stress conditions with β-amyloidosis, although further study is needed to understand the precise mechanisms underlying interactions between these pathways in AD.

In conclusion, the results presented here demonstrate that GCN2 deletion facilitates rather than suppresses phosphorylation of the translation initiation factor eIF2α through overactivation of another eIF2α kinase PERK and exacerbates BACE1 and ATF4 elevations in 5XFAD mice, resulting in the aggravation of β-amyloidogenesis and CREB dysfunction. Although further investigation is required for the demonstration of causal link, it is conceivable that PERK may be a crucial mediator of eIF2α phosphorylation responsible for pathogenic translational dysregulation under β-amyloidosis and thus represent a potential target for therapeutic interventions to treat AD.
